# Singing for the Rehabilitation of Acquired Neurogenic Communication Disorders: Continuing the Evidence Dialogue with a Survey of Current Practices in Speech-Language Pathology

**DOI:** 10.3390/healthcare10061010

**Published:** 2022-05-30

**Authors:** Estelle Behaghel, Anna Zumbansen

**Affiliations:** 1 Department of Speech-Language Pathology, Université de Limoges, 87036 Limoges, France; estelle.lecomte@etu.unilim.fr; 2 School of Rehabilitation Sciences, University of Ottawa, Ottawa, ON K1H 8M5, Canada; 3 Music and Health Research Institute, University of Ottawa, Ottawa, ON K1N 6N5, Canada

**Keywords:** singing, speech therapy, music therapy, communication disorders, stroke, dementia, professional practice, social prescribing

## Abstract

Therapeutic applications of singing (e.g., melodic intonation therapy) for acquired neurogenic communication disorders (ANCD) such as post-stroke aphasia, dysarthria, or neurodegenerative diseases have emerged from innovations by clinical speech-language pathologists (SLPs). However, these specialists have never been systematically consulted about the use of singing in their practices. We report a survey of 395 SLPs in France using an online questionnaire (September 2018–January 2019). Most (98%) knew that singing could be a therapeutic tool. A wide variety of uses emerged in our data. Some practices (e.g., song games) have not yet been investigated in research settings. Melodic therapy, which is supported by scientific evidence, is familiar to clinicians (90%), but they lack training and rarely follow a full protocol. Over half of respondents (62%) recognize group singing for various benefits, but do not often use it, mainly due to the lack of adapted or welcoming choirs in their area. These results provide key information for continued dialogue between researchers, clinicians, and the community. Considering the aging population and the associated increase in the prevalence of ANCD, access to group singing in particular could be facilitated for these patients from a social prescription perspective with further research evidence.

## 1. Introduction

Singing is a musical activity with high therapeutic potential in neurological rehabilitation, particularly in acquired neurogenic communication disorders (ANCD), such as aphasia or dysarthria caused by traumatic brain injury, stroke, or neurodegenerative diseases [1,2,3]. For example, melodic therapies based on sung speech have proven effective for language recovery in Broca’s aphasia [4,5]. Singing in a choir improves speech disorders in Parkinson’s disease and impacts the well-being and quality of life of participants [6]. Singing also improves several extralinguistic components with a high impact on communication disorders in dementia, including mood, orientation, long-term episodic memory, attention, executive function, working memory, and general cognition [7].

Singing may be of benefit due to its musical nature. Music therapy is used for a wide variety of health conditions, including ANCD. Therapeutic approaches using music have been organized in various frameworks. For example, the term Neurological Music Therapy is related to a purely neuroscientific framework, where therapies are classified according to the diagnostic treatment goal and the way music is used to achieve this goal in people with neurological diseases [8]. It is therefore readily applicable to ANCD. A more recent and broader framework includes the neuroscientific framework and further considers the effect of music therapy on the individual beyond their neurological deficit [9]. The dominant level, or mechanism of response, in this model is the learned cognitive response related to our emotional experiences with music. Music is a complex cognitive stimulus that enriches environmental stimulation, an essential factor in neurological recovery [10]. It is also considered useful in rehabilitation to improve motivation for intensive therapy through the effects of entrainment and by promoting a positive mood [11].

From a speech-language therapy point of view, singing is a form of music of particular interest for ANCD. It is performed with the same organs as speech, which entails a significant degree of sharing of peripheral and central neural resources. However, brain damage that causes a language disorder (aphasia) can spare musical abilities, allowing victims to sing [12]. Thus, singing has long been considered a motivating method for the rehabilitation of ANCD. Recent systematic reviews on music rehabilitation encourage further research with more controlled studies [1,2,3].

The singing-based interventions that have been studied in ANCD take various forms, with both group and individual interventions. Activities include singing warm-up exercises, such as physical, respiratory, phonatory, resonance, and articulatory exercises [13,14]. The musical material is comprised of songs (familiar or not) [15] or simple melodic and rhythmic lines composed from natural speech, which is typical of the melodic therapy approach, or Melodic Intonation Therapy (MIT) [16]. Overall, the literature focuses on two approaches that have now been examined in randomized controlled studies (RCT): (1) melodic therapy and its variants and (2) group singing (hereafter also referred to as “choir” or “choral singing”). Therefore, in this paper we will especially focus on melodic therapies and group singing in addition to singing-based interventions in general.

The characteristic of all melodic therapy approaches is having phrases of daily life uttered in a sung intonation following a simple melody [17]. In the original MIT [16,18], patients learn the intoned speech technique and then return gradually to normal speech while internalizing this way of speaking/singing to promote generalized language recovery. Its effectiveness is now supported by an RCT with 27 patients with aphasia at the subacute stage post-stroke [4]. So-called “palliative melodic therapies” [17] focus treatment on a few words or sentences, and patients practice these phrases repeatedly [19]. This contrasts with the original MIT, where the focus is on learning the technique and not memorizing specific sentences, which is avoided by training patients on numerous and varied sentences [17]. Palliative MIT versions offer patients with severe forms of expressive aphasia a goal that may be easier to achieve than generalized language recovery by providing them with ready-made sentences that may help them in their daily lives. The efficacy of palliative MIT is supported by an RCT in 30 patients with aphasia in the acute to subacute stage post-stroke [20] and by a non-randomized controlled group study in 15 chronic post-stroke aphasic patients [21]. Finally, another variation of MIT, Melodic and Rhythmic Therapy, or Therapie Mélodique et Rythmée (TMR), was developed in France by adapting the intoned speech technique to French, a syllable-timed language (cf. English as a stress-timed language), and by adding emphasis on the rhythmic aspect of the melody. In contrast to other melodic therapy approaches, there is no guided return to normal speech. With TMR, the patient learns to overtly use the intoned technique if needed during speech [22]. TMR has yielded promising results in a case series [23].

With regard to choral singing, 11 studies have been identified in a recent systematic review on the effect of this form of singing for the rehabilitation of neurological communication disorders, including Alzheimer’s dementia, post-stroke aphasia, and Parkinson’s disease [2]. Of these, nine studies report a significant improvement in at least one of the measures related to communication. This literature is marked by considerable methodological variability in terms of dosage (session duration: 50–120 min; session frequency: three per week to two per month; activity duration: 6 weeks to 2 years), session content (including or not including targeted voice and speech exercises; socializing breaks), choir leader qualification (in music, music teaching, voice and speech-language therapy, music therapy, and trained volunteers), and presence of a concert performance and homework in the protocols. The most reliable effect so far is an improvement in motor speech skills in Parkinson’s dysarthria demonstrated in a non-randomized control trial with 75 participants [6]. This protocol consisted of weekly or monthly 120 min choir sessions over three months, led by trained community musicians and volunteers and included specific voice exercises for Parkinson’s dysarthria as well as socializing breaks in addition to singing.

Melodic therapy and choir singing differ in several important practical ways. One is an individual therapy and the other takes place in a group setting. Moreover, melodic therapy is a speech-language therapy protocol specifically designed for the rehabilitation of aphasia, whereas group singing is usually not related to a therapeutic context but can be shaped by music therapists and other health specialists for different clinical purposes. For these reasons and for clarity, we will separate these approaches in this paper.

The variety of forms of song use in the scientific literature suggest an even wider range of practices in clinical settings. In fact, the studied approaches (including the melodic therapy approaches) emerged from innovations in the clinical field. According to the current conception of evidence-based practice [24], the opinions of clinicians are as important as the results of scientific studies. The clinical field can nourish research as much as the results of research support and inspire clinical practice. The clinical specialists in communication disorders are speech-language pathologists (SLPs). However, to our knowledge, they have never been systematically consulted about the use of singing in their practice with ANCD. Here, we report the results of a survey of SLPs in France. We sought to examine the knowledge and use of singing in clinical practice in neurological rehabilitation of communication disorders. We explored the use of singing, first without specifying its form, and then delved into the clinical practice of melodic therapy and choral singing.

## 2. Materials and Methods

### 2.1. Conception of the Study

We conducted an anonymous online survey from September 2018 to January 2019.

### 2.2. Target Population

All SLPs practicing in France (including overseas departments) were targeted, regardless of their mode of practice (private and/or public clinics) or the type of clinical population. Speech-language pathologists in France must hold a certificate that is awarded after five years of university training (Master’s degree) including 3158 h of coursework and 2040 h of internship. The training includes courses in linguistics, psychology, education, neuroscience, ENT, pediatrics, psychiatry, and practica in communication, cognition, oral and written language disorders, as well as speech, voice and swallowing disorders. There were 25,607 SLPs in France as of 1 January 2018 [25]. The respondents consented to participate in this study by completing the anonymous questionnaire. The ethical compliance of the project was approved by the University of Limoges.

### 2.3. Type and Size of the Sample

Because we could not obtain the contact details of all individuals in the population, strictly random sampling could not be carried out. We called on the voluntary participation of SLPs by distributing the survey as widely as possible among this population via the Internet. We determined our target number of respondents using the sample size calculator available at surveymonkey.com (accessed on 17 September 2018) by entering the population size of SLPs in France (25,607) and setting a 95% confidence level. We therefore aimed to obtain at least 379 responses to the survey.

### 2.4. Questionnaire

For this survey, we built an online questionnaire using the free service, Google Forms. A pilot version of the questionnaire was tested with three SLPs in order to identify and adjust wording that lacked clarity. We estimated that it could be completed within approximately 20 min. An English translation of the final version of the French-language questionnaire is available in the Supplementary Material. The questionnaire was divided into three independent parts: knowledge and use of (1) singing, (2) melodic therapy, and (3) choral singing as therapeutic tools in ANCD. Each part consisted of closed and semi-open questions (multiple choice including “Other” with a request for precision) and an open-ended question. Some questions were presented conditionally based on answers to previous questions using the redirection system included in the online survey platform, so not all respondents had to answer all questions. The number of responses for each question is reported in the results.

### 2.5. Procedure

The link to the online questionnaire was distributed on 7 September and again on 9 November 2018 to four Facebook groups that bring together French SLPs. We also sent messages to the email list of the speech-language therapy union from the Limousin region, the clinicians affiliated with the University of Limoges, and our personal network, asking clinicians to participate and to forward the message to other clinicians.

### 2.6. Data Analysis

The data were exported from the online survey platform to a table in Microsoft Excel for analysis. Answers to closed-ended questions were counted, translated as a percentage of responses, and represented in pie charts. The “Other” answers in the semi-open multiple-choice questions were read and grouped into categories. Most could be added to the two or three response categories already provided in the multiple-choice questions. To facilitate analysis, a category title was slightly adjusted: the category “I have not received training in therapy” in multiple-choice question #11 was adjusted to “I have not received sufficient training in melodic therapy”. Thus, some “Other” responses, such as that of participant P257: “I only received information about this therapy; I was not informed of the existence of a training that was sufficiently complete.”, were redirected to this adjusted answer category.

The open-ended answers were subject to thematic analysis. One of the authors (EB) read all of the responses several times and then extracted themes, which she grouped into categories. The other author (AZ) reread this coding and suggested changes that were adopted or not after discussion. A series of encoding and inference rules were established to make encoding reproducible within the laboratory (e.g., when a respondent mentioned using singing with dysarthric patients, we inferred that at least one of the therapeutic goals targeted speech). Three master’s students in speech-language therapy blindly recoded the participants’ responses according to these rules. Disagreements were discussed and resolved by consensus, sometimes providing necessary clarifications to coding rules. When “memory” was identified and coded as a therapeutic objective in a participant’s response, we inferred the type of memory (e.g., semantic, episodic, etc.) when possible and had it reviewed by an independent certified neuropsychologist.

## 3. Results

Four hundred and five people responded to the survey. Eight participants were excluded because they were not SLPs, and two duplicates were removed. We therefore analyzed 395 responses from SLPs. We present the results according to the three sections of the survey (singing, melodic therapy, and choral singing).

### 3.1. Singing

**Figure 1:**
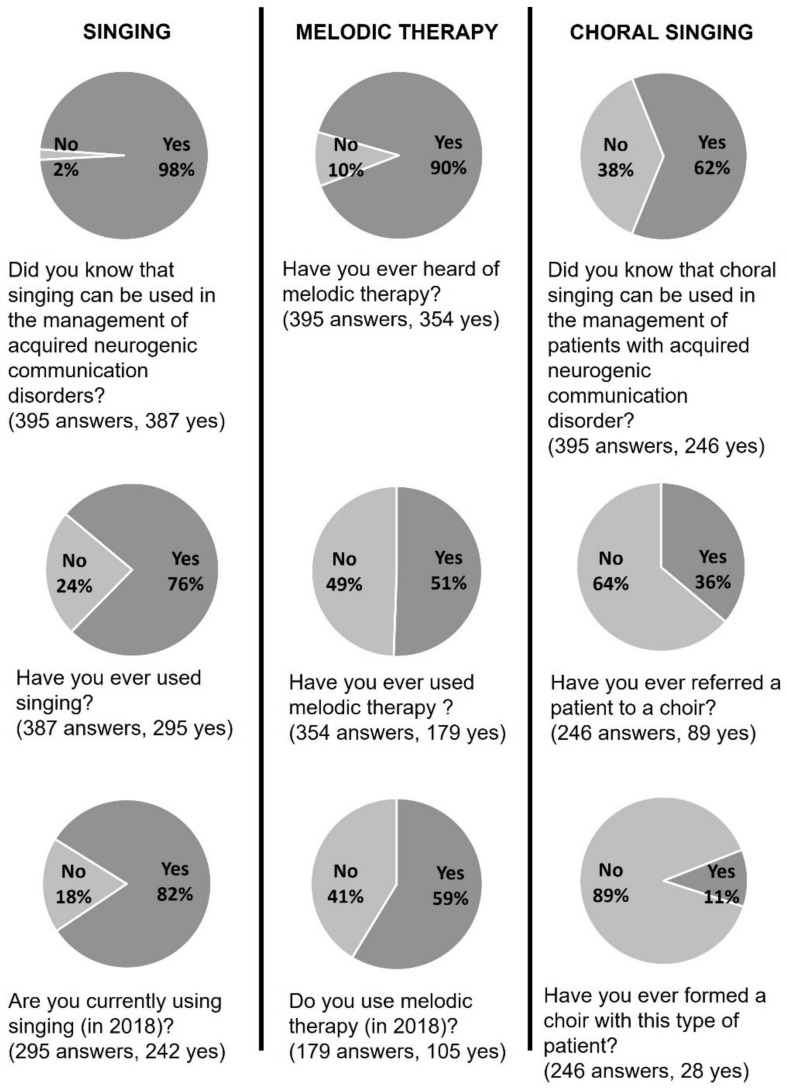
Answers to the closed questions of the questionnaire in the Singing, Melodic Therapy, and Choral Singing sections.

**Figure 2:**
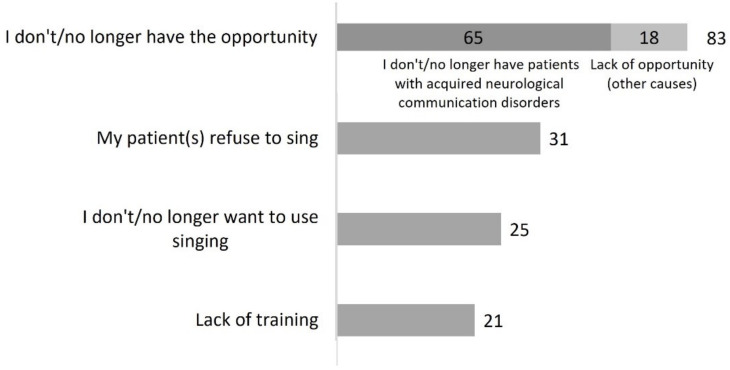
Reasons why participants do not/no longer use singing (question #6 of the questionnaire, 145 respondents).

Three hundred and eighty-seven SLPs (98%) reported knowing that singing can be used for the treatment of ANCD. The majority (*n* = 295, 76%) had used singing at some point, and of those, most were currently using singing (*n* = 242, 82%) (Figure 1). A total of 145 participants (37%) who were aware that singing can be a therapeutic tool did not use it (*n* = 92, 63%) or did not use it anymore (*n* = 53, 37%). As shown in Figure 2, most explained this by citing a lack of opportunity, which may correspond to a stoppage of professional practice (“Currently on sick leave”—P232) or to the absence of patients with ANCD in their caseload. Less common reasons were a refusal to sing from the patient or SLP. Finally, 21 respondents mentioned a lack of training.

Table 1 shows the thematic analysis of the 295 responses to the open-ended question: “Could you briefly explain how you used singing with these patients?”. Themes are presented by category (Disorders; Therapy formats; Targeted domains; Modalities; Activities; Types of music) in decreasing order of frequency. 

We identified 19 types of therapeutic activities in which singing was used in our sample. The most common were the completion of sung phrases (*n* = 50), melodic therapy (*n* = 49), singing in unison (*n* = 40), song games (*n* = 30), vocal exercises (*n* = 26), and listening (*n* = 21). The type of music was most often personalized (*n* = 80). Popular songs were more frequent (*n* = 62) than nursery rhymes (*n* = 17) or prayers (*n* = 2). Individual therapy was mentioned more often (*n* = 278) than group therapy (*n* = 46). Two respondents also reported assigning singing as homework to complement therapy sessions. 

SLPs gave a variety of reasons for using singing in therapy (15 target domains). The most frequent targets were those of language (*n* = 200), memory (*n* = 143, most often semantic memory), and speech and voice (*n* = 101). Executive function (*n* = 40), restoration of speech (*n* = 37), pleasure (*n* = 34), and rhythm (*n* = 28) were also mentioned quite frequently. Among 13 identified disorders, the most frequently cited were neurodegenerative diseases (*n* = 109, most often Alzheimer’s type dementia and Parkinson’s disease) and aphasia (*n* = 84).

### 3.2. Melodic Therapy

**Figure 3:**
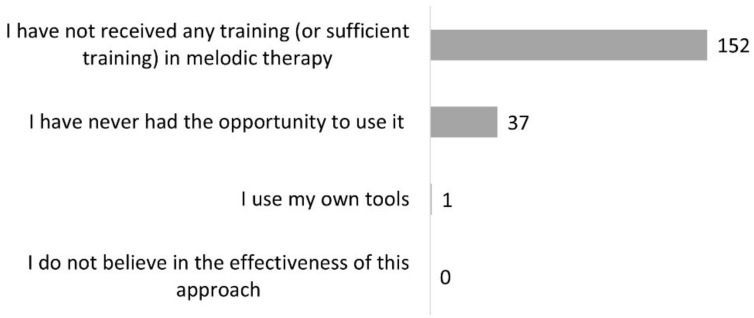
Reasons why participants have never used melodic therapy (question #11 of the questionnaire, 175 respondents).

**Figure 4:**
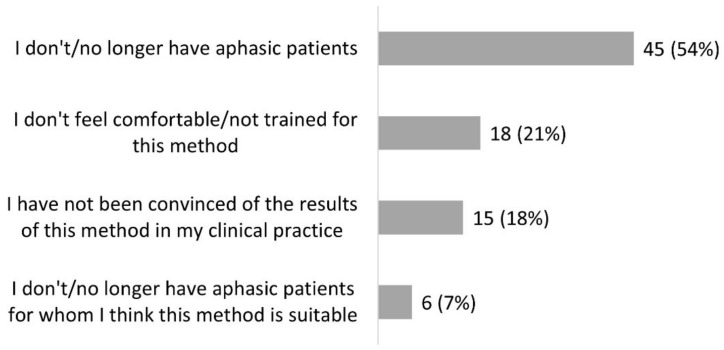
Reasons why participants no longer use melodic therapy (question #12 of the questionnaire, 74 respondents).

Three hundred and fifty-four SLPs (90%) had heard of melodic therapy. Half of those (*n* = 179, 51%) had already used it. Of this number, 105 (59%) still used it (Figure 1). Among the 175 SLPs who knew of the existence of at least one melodic therapy but had never used it, 152 (80%) reported insufficient training, and 37 (19%) said they had never had the opportunity to use it (Figure 3). Among the 74 clinicians who had stopped using it, 45 (54%) explained that they had no patients in the target population for this approach, 18 (21%) declared that they did not feel comfortable or sufficiently trained for this method, and 15 (18%) explained that they were not convinced by the results in their practice (Figure 4). If we sum the number of respondents who did not know of melodic therapy (*n* = 41) and the lack of training reported in Figure 3 and Figure 4, we obtain a total of 211 respondents, or 53% of SLPs, who do not have sufficient training to correctly apply melodic therapy.

Table 2 displays the thematic analysis of the 179 responses to the open-ended question: “Could you briefly describe how you incorporated melodic therapy into your practice?”. Similar to the previous table, the themes are presented by category (Sources of information; Disorders; Impairment severity; Targeted domains; Observance of the method; Departures from conventional programs; Time of use; Importance within therapy options) in decreasing order of frequency. 

Few clinicians reported using a complete melodic therapy program (*n* = 22). Twice as many declared that they only used the facilitation technique of the method (*n* = 45). Some said they used part of a melodic therapy program (*n* = 12), and there was mention of departures from published methods, such as pairing with regular singing (*n* = 2) or text reading (*n* = 2). Melodic therapy was often offered by SLPs when they first began working with a patient (*n* = 22). Twelve SLPs indicated that they used it at each session. Eighteen SLPs mentioned that melodic therapy was an important part of their therapeutic offerings, while others were using it among other methods (*n* = 11), rarely (*n* = 15), or as a second choice (*n* = 2).

We identified six types of target domains for the use of melodic therapy (Table 2), including language in 165 responses, speech restoration in 22, and speech in 12. When participants indicated in the open-ended answers that they used melodic therapies for specific disorders, aphasia was the most frequent disorder mentioned (*n* = 80) and, when specified, was of the non-fluent type (*n* = 32). Seven SLPs reported using melodic therapy with people with Parkinson’s disease or dysarthria.

Some clinicians specified their sources of information about the method. Personal reading was mentioned as much as formal training (*n* = 12). Only three clinicians mentioned being taught melodic therapy during their initial training. Eight clinicians declared that they had relied on consultations with colleagues.

### 3.3. Choral Singing

**Figure 5:**
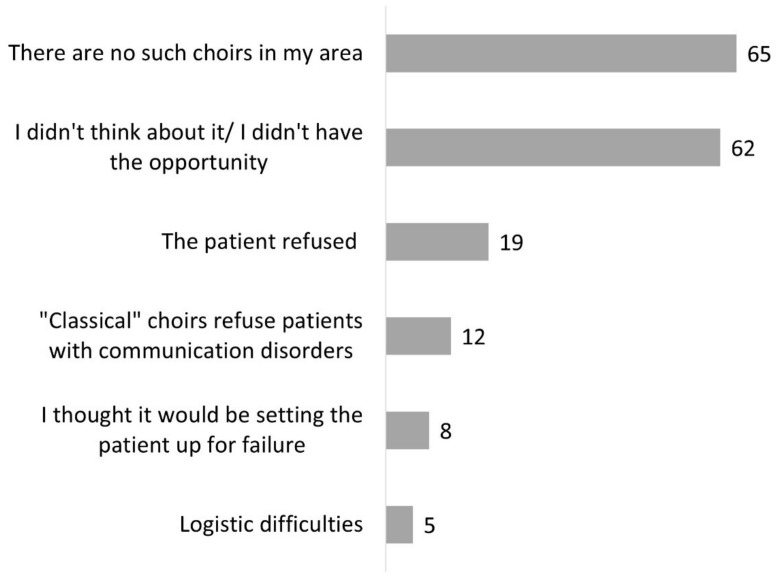
Reasons why participants did not refer patients to a choir (question #18 of the questionnaire, 157 respondents).

Two hundred and forty-six SLPs (62%) knew that choral singing could be used for ANCD (Figure 1). Among these clinicians, 89 (36%) had already referred a patient to a choir either adapted to this clinical population (*n* = 30), a general choir (*n* = 56), or both (*n* = 3). Clinicians who had not referred patients to a choir explained that there was no adapted choir in their region (*n* = 65) and/or they had not thought of it or had not had the opportunity (*n* = 62). It could also be a refusal on the part of the patient (*n* = 19) and/or the choirs who did not want to recruit people living with ANCD (*n* = 12) (Figure 5).

**Figure 6:**
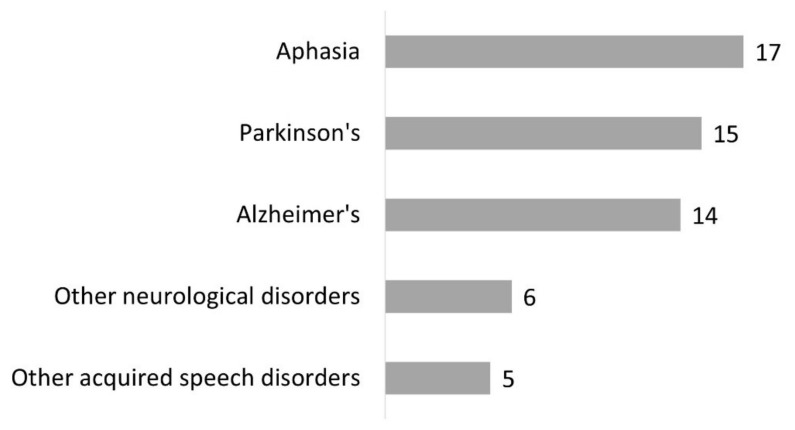
Type of disorders for which participants created a choir (question #16 of the questionnaire, 28 respondents).

A few of our respondents (*n* = 28) had created a choir for patients (Figure 1). They mentioned mainly patients with aphasia (*n* = 17), Parkinson’s disease (*n* = 15), and Alzheimer’s disease (*n* = 14) (Figure 6). Other neurological disorders (*n* = 6) and neurological speech disorders (*n* = 5) were also specified. Most often, SLPs indicated several types of disorders.

**Figure 7:**
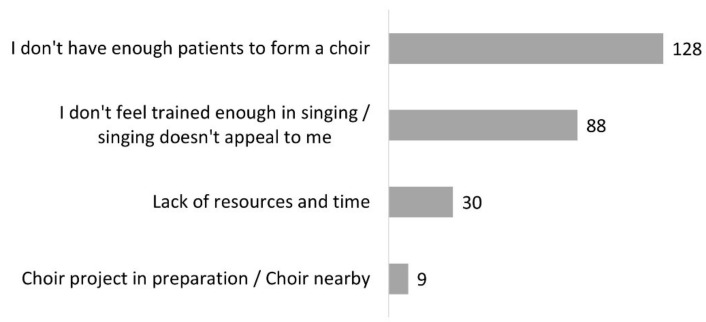
Reasons why participants did not start a choir (question #15 of the questionnaire, 218 respondents).

The 218 respondents who had never created a choir explained that they did not have enough patients to form one (*n* = 128), they lacked training in singing or singing did not appeal to them (*n* = 88), or they lacked time and means (*n* = 30). Nine respondents mentioned that a choir already existed or was planned (Figure 7). In summary, according to the data illustrated in Figure 5 and Figure 7, the reason why patients do not join choirs is less often because they refuse than that they do not have access to them.

Table 3 shows the thematic analysis of the responses to the open-ended question: “Could you briefly explain why you referred a patient to a choir?”. As before, the themes are presented by category (Targeted domains; Favorable conditions for the success of this referral; Complementary role to conventional speech-language therapy; Disorders) in decreasing order of frequency.

The targeted therapeutic objectives in referring a patient to a choir were speech (*n* = 34), social ties (*n* = 33), pleasure (*n* = 15), language (*n* = 14), well-being (*n* = 11), and more generally for its complementary role to conventional speech-language therapy (*n* = 16). Respondents also cited benefits for memory, cognitive functions, communication, and sense of identity. Eleven respondents specified that the referred patients had Parkinson’s disease. Only four of these clinicians had referred the patients to an adapted choir. Two respondents mentioned Alzheimer’s disease. These two SLPs referred patients in the early stages of the disease to a general choir for memory stimulation.

Clinicians also mentioned several favorable conditions for the success of their referral: the patient was a former chorister (*n* = 9), the benefits of singing were noticed during individual therapy sessions (*n* = 5), there was a nearby suitable and available choir (*n* = 5), the patient liked to sing (*n* = 3), and the request came from the patient (*n* = 3).

## 4. Discussion

The results of this survey draw an in-depth picture of the use of singing in the current practice of SLPs when dealing with patients with ANCD. Almost all of the respondents knew that singing, in any form, can be used (98%), slightly fewer were aware of melodic therapy approaches (90%), and just over half (62%) knew choral singing as a therapeutic tool for this clinical population.

### 4.1. Singing

Singing is a therapeutic tool commonly used by SLPs (76% of respondents) for ANCD. Our field data echo recent findings from several systematic reviews, which show the therapeutic potential of singing in neurological rehabilitation [1,2,3]. SLPs use singing primarily to work on speech, language, and memory, which is in line with data from controlled group studies. After a stroke, the addition of music-based therapy and vocal improvisation may increase the effects of speech-language therapy on various aspects of spontaneous language [26]. Listening to personalized music also helps recover cognitive functions, including language and memory, and promotes a positive mood [27]. Speech, language, and memory are typical treatment targets in the pathological conditions mentioned by our respondents: aphasia, neurodegenerative diseases, including dementias of the Alzheimer’s type and Parkinson’s disease, and dysarthria. Data from the literature do indeed show benefits of using music and singing for recovery from aphasia [13] and for people with neurodegenerative diseases (dementia and Parkinson’s disease) for mood, cognition, well-being, and quality of life [7,28,29]. The effect of singing for the treatment of dysarthria has been best demonstrated in Parkinson’s disease [6].

Interestingly, three of our respondents used singing for swallowing rehabilitation. We did not find any corresponding study in the literature. Likewise, clinicians used singing for different types of dysarthria, not limited to Parkinson’s dysarthria. These field data encourage further research into these topics.

SLPs recognize the positive effect that music can have on mood and arousal, and they use it to promote therapeutic bond and patient engagement in therapy. A total of 49 respondents mentioned that pleasure was one of the goals when using singing (*n* = 34) or choral singing (*n* = 15). The beneficial effect of listening to or singing personalized songs on mood is supported by controlled studies on stroke rehabilitation [27] and neurodegenerative diseases [7]. Mood enhancement has also been documented in several studies on therapeutic choirs with patients with ANCD [30,31,32].

Some of our respondents invoked singing as a support for maintaining a good therapeutic relationship (“I use singing, not as a tool, but when it comes at the right time during a conversation, an association of ideas, a particular mood, always with the idea of being connected with the patient.”—P264). Others mentioned the motivating effect of singing during therapy (“we work in an enjoyable atmosphere and with a pleasant tool. They don’t really feel like they are working and that makes it more fun and interesting”—P321). We also believe that music and singing help to maintain the therapeutic bond and the motivation in individual therapies that can sometimes be repetitive and laborious. The effect on motivation has not yet been tested, but has been suggested by several other researchers [11,33,34].

Finally, SLPs mentioned that music and songs are used to awaken their patients to communication (“singing or listening to music of their time: memory wakes up, (…) non-verbal expression intensifies, residents deeply affected come out of their silence”—P17). Numerous studies support the use of music therapy for arousal and overall stimulation, including in patients in a vegetative state [35,36]. In sum, the effect of singing on arousal, mood, motivation, and therapeutic bonding appears to be of great interest in the management of communication disorders. It would be important to continue interdisciplinary research in speech-language and music therapy and validate these effects in studies of high methodological quality. In the meantime, collaborations between SLPs and music therapists may be encouraged when possible (as there are currently only about 600 certified music therapists in France) [37].

Our results show that the form and content of therapy sessions involving singing are varied, as is the case in the literature [1,2,3]. It should be noted that our respondents referred to individual therapy (*n* = 246) more often than group therapy (*n* = 46), probably because the former is the “default mode” in speech-language therapy. Groups were mentioned in the context of choirs or groups within clinical institutions, but rarely in private practice (we will come back to this below). Sihvonen et al. [3] note in their review that few studies (other than those focusing on dementia) have used patient-preferred music. In contrast, our field data suggest that most clinicians select songs according to the preferences of the patient or from their well-known repertoire. Indeed, familiar songs can trigger automatisms on which some treatment approaches rely. Moreover, personalized music can facilitate patients’ collaboration through emotional engagement: it has been shown that music listened to during childhood and adolescence is better known than that of other stages of life and elicits stronger emotional involvement [38].

Completion of song phrases was the most frequently cited activity in our data (Table 1). Research supports the immediately rewarding effect of this activity because most people with aphasia are successful at it [39]. However, its long-term effects have yet to be examined. Melodic therapy and unison singing are the next most common singing activities mentioned by our respondents. These have been studied to an extent and will be discussed below, along with the survey sections dedicated to melodic therapy and choir practice. Finally, listening to songs is another activity that is frequent in our data and has been positive for patients in several studies, particularly for post-stroke recovery [27] and for relaxation of patients, even in a vegetative state [36].

Other musical activities (i.e., song games, mimes, reading, automatic series, and the psychophony method) used by SLPs have not been scientifically tested in ANCD. This contrast is striking for song games, which are mentioned 30 times in our results. These include melody quizzes and various association riddles between song lyrics, melodies, titles, and performers. Rehabilitation scientists could investigate the therapeutic potential of these cognitive games used in the clinic.

On the other hand, the use of musical instruments (such as percussion instruments) has been studied as part of several intervention protocols in language or cognition research [13,26,28] but were only mentioned twice by our respondents, perhaps because we did not ask them explicitly and they use them outside of singing activities. SLPs may, however, enrich their panel of singing intervention tools with simple instruments accessible to patients with ANCD.

Our data also show that singing is not appreciated by everyone, a point rarely made in the literature. Our participants reported that some patients and/or they themselves do not wish to sing (Figure 2, Figure 5 and Figure 7). Although it is a natural and universal activity in humans, singing is sometimes considered to be reserved for a trained elite, and can be experienced as more stressful than pleasant. How to bring the activity of singing in a non-threatening and fun way rests with the clinician. Music therapists are trained to do this. It is also possible that singing may be considered an inappropriate activity by some patients in a formal situation such as the traditional setting of speech-language therapy. Creative collaboration between SLPs and music therapists may help overcome this limitation. In the scientific literature, refusal to participate in a clinical trial on singing is not always reported. A few authors report the attrition rate, but these data are often incomplete [1]. It will be important to improve study reports in this regard, since people who dislike singing appear to be not uncommon.

We also noticed that singing can be enjoyed, but not in all its forms. For example, one respondent felt that melodic therapy “has nothing to do with singing” (P305). Other SLPs regret the unnatural aspect of melodic therapy (“It didn’t quite appeal to me, because of the very ‘mechanical’ aspect; that being said, it does work with some patients; I prefer voice and singing because it is much livelier!”—P9). Choir practice could also be a form of singing that is not appreciated. For example, patients might not enjoy group dynamics or the idea of a choir. However, as we explain below, our results indicate that, currently, lack of access to choirs is a much more significant reason than refusal for non-participation. Whatever the reasons, the few cases of refusal that were reported by our respondents illustrate the importance of considering the values and preferences of the patient-clinician dyad in evidence-based practice [24].

Overall, our results show that the vast majority of SLPs who do not use singing (in any form) do not do so for three reasons: they do not have the opportunity (this includes the absence of the target population in their caseload); they feel they lack training; or they did not know that singing could be used in therapy (2% of respondents).

### 4.2. Melodic Therapy

Almost half of the surveyed SLPs have already used melodic therapy at least once. The ones who know of this treatment approach without having ever used it mentioned above all a lack of sufficient training.

Most often, clinicians reported using melodic therapy for language and speech restoration in patients with severe non-fluent aphasia, which corresponds to the clinical profile targeted by the designers of melodic therapies [17]. Authors of the original MIT also clarified that patients with good repetition skills (i.e., non-fluent aphasia of the transcortical motor type) were poor candidates for the method [40]. This was not reflected in the responses to our survey. A few respondents also cited other clinical profiles, such as Parkinson’s disease and dysarthria, that they used melodic therapies for, as well as employing it for purposes other than speech or language, such as sense of identity and rhythmic abilities. These uses of melodic therapies have not been investigated in research to date.

Our results reveal that clinicians rarely follow an entire melodic therapy program (*n* = 22 only), and rather use the intoned speech technique in isolation (*n* = 45) (“I used this rhythmic and oral intonation expression to help patients initiate or cue their productions”—P117). This finding could be linked to the lack of training pointed out above. However, SLPs can grasp the essential elements of melodic therapy and use them intuitively with their clinical expertise. The addition of aspects not typical of melodic therapies, such as association with regular singing (*n* = 2) or in a text reading activity (*n* = 2), supports the idea of a certain creativity of therapists within the approach while keeping the facilitation technique as a starting point. Some participants specified that they used parts of a melodic therapy program, possibly because the intensive dosage that is recommended is difficult to apply in usual clinical practice.

SLPs have sometimes stopped using melodic therapy because they were not convinced by the results of the method. It is interesting to try to understand these cases. MIT is one of the few treatment programs in speech-language therapy supported by an RCT [4]. In this trial, the proposed therapy was the original MIT delivered at the intense recommended dosage (at least 3 h/week with a trained clinician), and the study was carried out with 27 patients in the subacute post-stroke stage having the clinical profiles of good MIT candidates. Given the lack of training of many clinicians, as revealed in our results (at least 53% of SLPs may not have sufficient training to properly apply melodic therapy), we think that some of the disappointed SLPs might have applied melodic therapy without meeting the necessary conditions for its success. For example, the intoned technique can have an immediate facilitation effect in patients, which is rewarding, but it does not guarantee a generalization of the effect. Without following the entire program at the recommended dosage, the facilitation effect is likely to remain confined to the moment when the speech is intoned with the active help of the clinician, which can be disappointing after a series of sessions. Most of the respondents who said they were not convinced of the results of the approach unfortunately did not explain whether a full melodic therapy program was followed. In one case, the response shows that only the facilitation technique was used (“offer the patient a model with melody, to be repeated”—P265), and in another, the respondent argues that it is practically impossible to apply the indicated dosage (“Not conclusive because in private practice, therefore not intensive”—P366). Finally, SLPs in France are better equipped to apply TMR than the original MIT because there is a manual and software in French for TMR but not for MIT. TMR would therefore be used more than MIT in France. The efficacy of TMR has not been established by any controlled group study to date.

In summary, our survey results reveal a need for training in melodic therapy approaches, which would be important to reduce the gap between current scientific evidence and practice. Meanwhile, further research on the efficacy of different melodic therapy variations is needed.

### 4.3. Choral Singing

Choral singing was less well-known as a tool in the rehabilitation of ANCD (62% of respondents) than other forms of singing, and few clinicians actually use it. When they do, they refer patients to an existing choir or create a choir themselves (Figure 1). Choir practice has been studied for its therapeutic effects much more recently [41] than melodic therapies, which date from the early 1970s [16]. This form of singing practice is worth exploring further in acquired communication disorders because it opens up the possibility of benefiting not only from the effects of singing, but also from the positive social effects of participating in a group [32,42]. As such, choral singing may be a valuable option of social prescription, a method whereby clinicians formally recommend sources of support within the community to patients to help them improve their health and well-being [43]. A first systematic review of the literature was published last year on the effects of choral singing in ANCD [2], which testifies to the growing interest of researchers in this approach.

Most of our respondents who were aware of rehabilitative benefits of choral singing were unable to refer patients to an adapted choir for patients because there was none in their area. There are indeed few choirs of this type in France: 39 according to a recent census, [44]. In contrast, there are many “regular” choirs (65,600 according to the European Choral Association, [45]), but our respondents reported that these groups are not always open to people with ANCD (Figure 5). A lack of knowledge about these neurological conditions and possible adaptations might explain, at least in part, the reluctance of some choirs. We believe that raising the awareness of regular choirs to communication disorders could open more opportunities for patients. More of these options would be a significant benefit because the creation of a dedicated therapeutic choir is a challenge. An adequate number of patients must be available, and it requires time, skills, and means that are not recognized by all health-care systems (Figure 7). However, it is encouraging to note that nine of our respondents indicated that such a choir already exists or is in the planning stages.

The therapeutic goals targeted by SLPs when they refer a patient to a choir are most often related to speech and social ties (Table 3). Interestingly, speech was more often targeted than language, while the reverse was true in the other parts of the survey on any form of singing or melodic therapy. Further, in contrast with the other survey sections, Parkinson’s disease, in which the communication disorder is greatly related to diminished speech motor skills, was the clinical profile most cited in the open answers (“especially interesting for Parkinson’s patients”—P371; “patients with Parkinson’s disease in order to work on rate, articulation, speech but also in a social approach”—P35). This suggests that clinicians think more of choir practice for Parkinson’s disease than for other pathologies, which is consistent with the current evidence on the effects of choir singing in ANCD [2]. A recent controlled study with 75 participants showed an effect on vocal intensity levels, which is typically too low in Parkinson’s dysarthria [6]. A pilot study with 10 participants also indicated an improvement in speech intelligibility, which is of great relevance for the daily life of these patients [46]. Monroe et al. [2] rightly note that the homogeneity of the speech disorders in this disease makes it easier to form groups of patients, including adapted choirs. However, this practical consideration cannot entirely explain our findings, since most respondents who referred a patient with Parkinson’s disease to a choir indicated a “general” choir. Choirs exclusively oriented toward people with Parkinson’s disease are rare in France (eight choirs according to Leguédé in 2018 [44]). Our data suggest the possibility that this clinical population could blend into general choirs quite easily.

Data from the literature encourage further consideration of choir practice for other pathologies, including Alzheimer’s type dementia [47] and post-stroke aphasia [48]. Only two respondents to our survey said they had referred patients with Alzheimer’s dementia in the early stage to a general choir for memory stimulation. For several clinicians, choir practice was seen to have a complementary role to classical speech-language therapy, making it possible to work on language, speech, or memory in a pleasant, social, and natural setting. Respondents mentioned benefits for well-being, memory, cognitive functions, communication, and sense of identity. The idea of complementarity with classical speech-language therapy is also mentioned by Särkämö et al. [7], for whom participation in a choir provided strong support for individual therapy in dementia. According to our field data, referral of a patient to a choir is easier if the patient is a former chorister, if there is an adapted choir nearby, if benefits of singing are noticed, and if the patient likes singing. The request for participating in a choir sometimes comes from patients themselves. As research advances and the potential benefits of choir practice are more widely known, it is likely that more clinicians will consider this therapeutic option for their patients.

### 4.4. Limitations

We did not carry out a strictly random sampling of the population of SLPs, and we did not collect demographic or professional data. The participants who answered the questionnaire were volunteers, probably for the most part already interested in our subject. Nevertheless, we obtained the participation of a high number of SLPs (exceeding the target number of responses by 4%), and the number of respondents unaware of melodic therapy (*n* = 41) or of singing as a tool for therapy (*n* = 8) indicates that we were able to obtain responses from people unfamiliar with our topic. We also note that in the absence of this sampling bias, we would expect to obtain an even higher percentage of SLPs not trained in melodic therapy and a lower percentage of respondents knowing about and using choir singing for therapeutic purposes. This would strengthen our conclusions about the need for training of SLPs in these two forms of therapeutic singing that are supported by scientific evidence.

It should also be noted that this survey was conducted in a single country. It is unknown to what extent clinical practice in France matches practices in other countries around the world, where SLPs might have different training and practice settings (e.g., over 80% of French SLPs work in private SLP clinics [25] where services are largely supported by the public health care system). Nevertheless, any potential sampling bias does not prevent us from identifying several significant research questions to be examined in the field of singing for ANCD.

## 5. Conclusions

This survey shows that singing is a therapeutic tool known by the majority of SLPs in France and is used in a wide variety of ways for the rehabilitation of ANCD. Some clinical practices identified by our participants have not yet been investigated within a research framework. Melodic therapy is familiar to clinicians, but they lack training and rarely follow a full protocol. Our data clearly show a need for more training opportunities in this speech-language therapy approach, which is supported by scientific evidence. Choral singing is less known as a therapeutic tool by our respondents and not as often used, mainly due to the lack of adapted or welcoming choirs in their area. However, research on the benefits of choir singing is developing rapidly and could spread in communities and clinical settings along with the popularization of social prescribing among clinicians. We believe this avenue takes on particular importance with the aging population and the related increase in the prevalence of ANCD.

## Figures and Tables

**Table 1:** Thematic analysis of open-ended answers to question 4: “Could you briefly explain how you used the singing tool with these patients?” (295 answers).

Theme				Occurrences	Quote (Example of a Participant)
Disorders					
	Neurodegenerative diseases (total)			109	
		Alzheimer’s type dementia		50	“with patients with Alzheimer’s disease, we sing familiar songs together.” (P293)
		Parkinson’s disease		44	“voice therapy with Parkinson’s patients” (P111)
		Neurodegenerative diseases		11	“as part of the rehabilitation in neurodegenerative diseases” (P62)
		Huntington’s disease		2	“targeting articulation and rhythm ((...) Huntington’s chorea...)” (P153)
		Multiple sclerosis		1	“I sometimes make people sing or hum (...) with MS” (P354)
		Progressive supranuclear palsy		1	“I recently received a patient with PSP and singing became my working tool with her” (P81)
	Aphasia (total)			84	
		Aphasia		81	“with an aphasic patient, I try to sing songs with her” (P168)
		Anomia		3	“using intonation in anomia therapies” (P46)
	Dysarthria			22	“In the context of dysarthria it works with the rhythm and the melody.” (P345)
	Voice disorder			5	“Vocalizations for voice rehabilitation.” (P346)
	Dysphagia			2	“Individual and collective for (...) dysphagia” (P34)
	Apraxia			2	“work of buccofacial apraxia” (P106)
	Neurological diseases			2	“I sing with my ‘neuro’ patients” (P231)
	Stuttering			1	“Use of nursery rhymes/songs for patients who stutter” (P373)
	Oral language disorders			1	“I have always used singing in all my rehabilitations (oral or written language)” (P13)
	Written language disorders			1	“I have always used singing in all my rehabilitations (oral or written language)” (P13)
	Deafness			1	“Constitution of a choir (with small orchestra) for people with cochlear implants” (P373)
	Ataxia			1	“Rhythm (in the context of ataxia)...” (P372)
Therapy formats					
	Individual			278	“Use of singing in one-on-one session” (P332)
	Group			46	“choral for people with aphasia” (P122)
	Homework			2	“at home as complementary work” (P72)
Targeted domains					
	Language			200	“Using automatic abilities to access language” (P47)
	Memory (total)			143	
		Semantic memory		79	“I hide the text and ask them to find the last word of each sentence” (P287)
		Memory		40	“Stimulating memory” (P90)
		Procedural memory		26	“ song lyrics used for the automatic ending of sentences” (P256)
		Autobiographical memory		19	“We also listen to music to bring out emotions and even memories” (P206)
		Episodic memory		10	“ to train episodic memory “ (P370)
	Speech and voice			101	“(...) with people who have lost in fluidity of speech” (P224)
	Executive functions			40	“I often propose songs to stimulate language, memory, attention, respect of rhythm” (P376)
	Speech restoration			37	“to restore speech in patients” (P120)
	Pleasure			34	“Sometimes also just for the pleasure of listening to an artist they liked” (P324)
	Rhythm			28	“by proposing (...) melodies to work on articulation and rhythm” (P153)
	Communication			18	“non-verbal communication intensifies” “working on turn taking “ (P232)
	Emotions			14	“expression of emotion through singing in someone who is disoriented” (P367)
	Awakening			9	“We would choose a song with the patient and (...) I would sing and the patient would follow or wake up to the words “ (P243)
	Sense of identity			7	“We sing together because this memory is preserved so it is gratifying” (P178)
	Cognitive stimulation			6	“The lyrics of the songs are the starting point for many cognitive stimulation exercises” (P88)
	Therapeutic bond			5	“I use singing (...) with the idea of being connected to the patient” (P264)
	Swallowing			3	“Rehabilitation of swallowing” (P94)
	Social ties			3	“A patient with Alzheimer’s disease participates in a choir, which is very valuable for him because it (...) gives him the opportunity to socialize” (P303)
Modalities					
	Production			241	“Singing duet with patient or music found on the internet and the patient sings the lyrics” (P74)
	Reception			241	“Listening to songs and working on emotions and memories” (P271)
Activities					
	Completion of sung phrases			50	“by playing musical excerpts to patients and (...) asking them to sing the rest. “ (P4)
	Melodic therapy			49	“use of the principles of Melodic Intonation Therapy or *Thérapie Mélodique et Rythmée*” (P234)
	Singing in unison			40	“ singing in duet with the patient” (P74)
	Song games (total)			30	
		Melody quizzes (total)		16	
			Melody quizzes	8	“finding the singer, or the title of a hummed song”, “*blindtest*” (P304)
			Melody/performer association	3	“humming a melody and the patient must find the title or performer” (P150)
			Melody/lyrics association	3	“beginning of melodic phrase… it’s up to her to put the lyrics to it” (P65)
			Melody/title association	2	“humming a melody and the patient must find the title or performer” (P150)
		Lyrics/song association		6	“search for songs from a word” (P174)
		Title/singer association		5	“association of a song title and its singer for example” (P239)
		Song games		3	“we make singing games” (P231)
	Vocal exercises			26	“warm-up vocalization (…) that will help to work on prosody” (P153)
	Listening			21	“Listening to songs” (P271)
	Choir			19	“singing group as part of a long-term care service in the hospital” (P339)
	Evocation			19	“pleasure to sing at the end of the session and remember the memories it evokes” (P150)
	Reading			15	“reading aloud the lyrics” (P365)
	Repetition			11	“singing phrases to facilitate their repetition”(P229)
	Karaoke			10	“when possible, I do karaoke” (P306)
	Rhythmic exercises			8	“we also work on purely rhythmic exercises” (P99)
	Body exercises			7	“I combine body and vocal exercises” (P9)
	*Psychophony* approach			3	“I use the tools and the approach of the *psychophony* method” (P391)
	Automatic series			3	“I use singing to initiate, recite, remember automatic series” (P224)
	Associated with the Lee Silverman Voice Treatment program			3	“As a complement to the LSVT” (P249)
	Beatboxing			1	“use of human beatboxing techniques” (P275)
	Mimes			1	“I sing mime rhymes (I sing and mime)” (P319)
Types of music					
	Personalized music			80	“use of songs known to the patient” (P232)
	Popular songs			62	“traditional French songs, *La Marseillaise*” (P131)
	Rhymes			17	“mainly in known nursery rhymes” (P291)
	Prayers			2	“ canticle heard many times in his childhood” (P263)

**Table 2:** Thematic analysis of open-ended answers to question 16: “Could you briefly describe how you incorporated the melodic therapy approach into your practice?” (179 answers).

Theme				Occurrences	Quote (Example of a Participant)
Sources of information					
	Training (total)			12	
		Continuous education		6	“I trained with Dominique Benichou last year.” (P78)
		Initial training		3	“I had some knowledge of this practice during my initial training” (P155)
		Training		3	“systematic protocol after training” (P69)
	Personal readings (total)			12	
		Manual		6	“In one-on-one session, with the help of the manual” (P34)
		Articles (total)		3	
			Van Eeckhout writings	2	“with texts by Philippe Van Eeckhout” (P177)
			Article	1	“I relied on a book or article describing this work” (P63)
		Software		2	“I have a software and I follow it” (P317)
		Personal reading		1	“I read a lot on the subject to deepen my understanding and practice” (P379)
	Colleagues			8	“I worked with a colleague who was trained; she introduced me to it a little” (P9)
	No training			2	“Unfortunately, I haven’t undergone the training yet” (P60)
Disorders					
	Aphasia			80	
		Aphasia		39	“with aphasic patients” (P217)
		Non-fluent aphasia		32	“early therapy in patients with non-fluent aphasia” (P167)
		Anomia		5	“in anomia therapies” (P46)
		Fluent aphasia		1	“sometimes in fluent aphasia” (P202)
		Mixed aphasia		1	“Mixed aphasia profile” (P349)
		Motor aphasia		1	“mainly in the context of motor aphasia “ (P355)
		Agrammatism		1	“With aphasic (…) agrammatic patients “ (P374)
	Parkinson’s disease			5	“with patients with (…) Parkinson’s disease to work on prosody” (P275)
	Dysarthria			2	“for dysarthric patients (…) with speech rate and prosody disorders “ (P6)
	Neurological diseases			1	“with aphasic or ‘neuro’ patients” (P98)
	Alzheimer’s type dementia			1	“with Alzheimer’s patients (…)” (P275)
	Developmental disorders			1	“ Whether it’s for (…) children with disabilities or aphasia, it’s always a good help” (P104)
	Language delays/disorders			1	“Whether it’s for big language delays (…), it’s always a good help” (P104)
Impairment severity					
	Severe			6	“I have used TMR with some patients with severe aphasia” (P38)
	Moderate			1	“For lexical production deficits in moderate aphasia” (P338)
Targeted domains					
	Language			165	
		Language		102	“In the context of massive aphasia, where automatic language can be spared” (P136)
		Language production		63	“For lexical production deficits in moderate aphasia” (P338)
	Speech restoration			22	“speech restoration in the beginning of care” (P7)
	Speech			12	“support for the articulation of a word” (P162)
	Rhythm			3	“I work on rhythm reproduction” (P119)
	Communication			2	“especially for all polite forms, communication openings, simple requests. “ (P251)
	Speech perception			1	“to work on auditory discrimination” (P188)
	Sense of identity			1	“It allows them to hear themselves, be proud” (P374)
Observance of the method					
	Isolated facilitation technique			45	“I chant the syllables following the melodic pattern” (P25)
	Entire program			22	“following the systematic protocol after training” (P69)
	Part of the program			12	“I have never been able to get past the rhythmic stage of the program because my patients are struggling with it” (P30)
Departures from conventional programs					
	Combination with more regular singing			2	“working on speech and communication disorders in association with pure singing” (P35)
	Reading			2	“Sentence repetition, sentence generation, reading in patients with dysarthria and aphasia “ (P345)
	Telling about one’s day			1	“We tell the patient’s day in this way: the lunch menu, the weekend activities...” (P40)
	Naming			1	“during naming exercises, with the support of syntactic constructions” (P70)
	Homework			1	“Modeling phrases to be trained at home” (P344)
Time of use					
	Beginning of care			22	“in the initial phase of aphasic patient’s care for speech restoration “ (P68)
	Each session			12	“I use it every session with patients with severe expression deficits” (P40)
	At the beginning of sessions			1	“at the beginning of each session and sometimes throughout the session in cases of very severe aphasia “ (P215)
	At the end of a session			1	“Quite often at the end of a session when fatigue sets” (P30)
Importance within therapy options					
	Important			18	“She is at the center of four of my interventions” (P31)
	Rare			15	“I use it too little to talk about it” (P99)
	Used among other approaches			11	“with people with aphasia (combined with other approaches) “ (P220)
	Second choice			2	“I have used TMR with some patients with severe aphasia after working with more traditional approaches” (P38)

**Table 3:** Thematic analysis of open-ended responses to question 19: “Could you briefly explain why you referred a patient to a choir?” (89 answers).

Theme		Occurrences	Quote (Example of a Participant)
Targeted domains			
	Speech and voice	34	“To free his voice, (…) stimulate rhythm and voice projection” (P393)
	Social ties	33	“to regain a social link” (P297)
	Pleasure	15	“because I thought it was very important for the patient to rediscover the pleasure of singing in a group” (P167)
	Language	14	“Because his language is more fluent in singing” (P25)
	Well-being	11	“Continuity of the work done in speech-language therapy with the addition of a social, playful and well-being dimension” (P312)
	Memory	8	“Patient in the early stage of Alzheimer’s disease seeking to maintain memory” (P304)
	Communication	5	“It depends, it can be for a rather functional or rather communicational purpose, or mixed more often!” (P220)
	Sense of identity	4	“Young patient (stroke) formerly a singer, with experience in choral singing, needing to restore her self-image and skills (…)” (P388)
	Cognitive functions	2	“(…) Cognitive and language stimulation” (P5)
Favorable conditions for the success of this referral			
	Former choir singer	9	“these were patients who had already practiced choral singing before and who had stopped because of their disorders” (P177)
	Singing benefits noticed	5	“Patient (…) showing clear signs of progress when using singing during sessions” (P12)
	Available adapted choir	5	“Towards a choir set up by the clinic where I worked, in collaboration with France Parkinson (…)” (P43)
	Likes to sing	3	“The patient already had an interest in singing, and liked to sing, so we thought that this would be a good activity” (P239)
	Patient’s request	3	“Because patients were asking for more singing and had trouble doing it alone (…)” (P33)
	Facilitated inclusion	2	“because I am part of this choir and it was easy for me to include them and enjoy the benefits of socialization” (P99)
	Easy to set up	1	“Because easily set up” (P127)
	Sufficient recovery	1	“It was a patient (…) who had progressed enough to feel comfortable again in a small country choir” (P229)
Complementary role to conventional speech-language therapy		16	“(…)to complete/enrich the care” (P73)
Disorders			
	Parkinson’s disease	11	“in a patient with Parkinson’s disease, to maintain skills acquired in rehabilitation” (P76)
	Alzheimer’s disease	2	“For a young patient with Alzheimer’s disease in the early stage, the goal is to stimulate memory (…)” (P75)

## Data Availability

The data presented in this study are available on request from the corresponding author.

## Supplementary Materials

The following supporting information can be downloaded at: https://www.mdpi.com/article/10.3390/healthcare10061010/s1, English translation of the French-language questionnaire.
