# Supplementary material for: Singing for the Rehabilitation of Acquired Neurogenic Communication Disorders: Continuing the Evidence Dialogue with a Survey of Current Practices in Speech-Language Pathology
Source: Healthcare (Basel). 2022 May 30;10(6):1010. doi: 10.3390/healthcare10061010 (PMC9222374; doi:10.3390/healthcare10061010)
Supplement: Supplementary file 1 [file healthcare-10-01010-s001.zip › healthcare-1691145-supplementary.pdf]

Singing for the rehabilitation of acquired neurogenic communication disorders: continuing the evidence dialog with a survey of current practices in speech-language pathology

-QUESTIONNAIRE FOR SPEECH-LANGUAGE PATHOLOGISTS-

1. Do you work, or have you worked, as a speech therapist?
- Yes
- No
- Other

Singing as a rehabilitation tool

2. Did you know that singing can be used in the management of acquired neurogenic communication disorders (e.g. aphasia, Alzheimer's-type dementia, Parkinson's disease...)?
- Yes
- No *(skip to question 7)*

3. If so, have you ever used singing in your practice with these patients?
- Yes
- No *(skip to question 6)*

4. Could you briefly explain how you used singing with these patients?

(open-ended answer)

5. Are you currently using singing (in 2018)?
- Yes *(skip to question 7)*
- No

6. If not, why ?
- I don't/no longer have patients with acquired neurogenic communication disorders
- My patient(s) refuse to sing
- I don't/no longer want to use singing
- Other

We are interested in melodic therapies

7. Have you ever heard of a melodic therapy (such as Melodic Intonation Therapy, MIT, in English, or Thérapie Mélodique et Rythmée, TMR, in French)?
- Yes
- No *(skip to question 13)*

8. Have you ever used it?

- Yes
- No *(skip to question 11)*

9. Could you briefly describe how you incorporated melodic therapy into your practice?

(open-ended answer)

10. Do you use it (in 2018)?
- Yes *(skip to question 13)*
- No *(skip to question 12)*

11. If not, why?
- I have not received any training in melodic therapy
- I do not believe in the effectiveness of this approach
- I have never had the opportunity to use it (e.g., few or no patients with neurogenic communication disorders)
- Other

(skip to question 13)

12. If not, why?
- I don't/no longer have aphasic patients
- I don't feel comfortable/trained for this method
- I have not been convinced of the results of this method in my clinical practice
- Other

We are particularly interested in choral singing

13. Did you know that choral singing can be used in the management of patients with acquired neurogenic communication disorders?
- Yes
- No *(skip to question 20)*

14. If so, have you ever formed a choir with this type of patient?
- Yes *(skip to question 16)*
- No

15. If not, why?
- I don't have enough patients to form a choir
- I don't feel trained enough in singing/ singing doesn't appeal to me
- Other

(skip to question 17)

16. If so, what types of disorders did patients have?
- Aphasia
- Parkinson

- Alzheimer’s
- Other

17. Have you ever referred a patient to a choir of this ilk or to a choir not specifically addressed to people with a disability (general choir)?

- Yes, to a choir of this ilk *(skip to question 19)*
- Yes, to a general choir *(skip to question 19)*
- No
- Other

18. If not, could you tell us why?

- There are no such choirs in my area
- The patient refused
- "Classical" choirs refuse patients with communication disorders
- Other

19. Could you briefly explain why you referred a patient to a choir?

(open-ended answer)

20. Thank you!
